# The influence of baseline characteristics, treatment and depression on health-related quality of life in patients with multiple myeloma: a prospective observational study

**DOI:** 10.1186/s12885-022-10101-9

**Published:** 2022-10-03

**Authors:** Julia Fischer, Stefan Knop, Sophia Danhof, Hermann Einsele, Daniela Keller, Claudia Löffler

**Affiliations:** 1 Department of Pediatrics, Wuerzburg University Medical Center, Josef-Schneider-Straße 2, 97080 Würzburg, Germany; 2 Department of Haematology and Oncology, Wuerzburg University Medical Center, Oberdürrbacher Straße 6, 97080 Würzburg, Germany; 3 “Daniela Keller – Statistik und Beratung”, Prosselsheimer Straße 4, 97273 Kuernach, Germany

**Keywords:** Multiple myeloma, Quality of life, Participation in clinical trials, Depression, Observational

## Abstract

**Background:**

Multiple myeloma (MM) is the third most common hematologic malignancy with increasing importance due to improving treatment strategies and long-term outcomes in an aging population. This study aims to analyse influencing factors on health-related quality of life (HRQoL), such as treatment strategies, participation in a clinical trial and patient characteristics like anxiety, depression, gender, and age. A better understanding of the individual factors in context with HRQoL could provide a helpful instrument for clinical decisions.

**Methods:**

In this prospective observational study, the HRQoL of MM patients with different therapies (first-line and relapse) was quantified by standardized questionnaires (EORTC QLQ-C30 and -MY20) in the context of sociodemographic data, individual anxiety and depressiveness (PHQ-4), and a selected number of clinical parameters and symptoms at defined time-points before, during, and after therapy.

**Results:**

In total, 70 patients were included in the study. The median age of the study cohort was 62 years. 44% were female and 56% were male patients. More than half of the patients were fully active with an ECOG 0. Global health status was significantly higher in patients with first-line treatment and even increased after start of therapy, while the pain level decreased. In contrast, patients with relapsed MM reported a decreasing global health status and increasing pain. Additionally, there was a higher global health status in less anxious/depressive patients. HRQoL decreased significantly after start of chemotherapy in the parameters body image, side effects of treatment, and cognitive functioning. Tandem stem-cell transplantation was not found to be a risk factor for higher impairment of HRQoL. Participation in a clinical study led to an improvement of most aspects of HRQoL. Among others, increased anxiety and depression, female gender, older age, impaired performance status, and recurrent disease can be early indicators for a reduced HRQoL.

**Conclusion:**

This study showed the importance of regular longitudinal assessments of patient reported outcomes (PROs) in routine clinical care. For the first time, to our knowledge, we were able to demonstrate a potential impact between participation in clinical trials and HRQoL. However, due to frequently restrictive inclusion criteria for clinical trials, these MM patients might not be directly comparable with patients treated within standard therapy concepts. Further studies are needed to clarify the relevance of this preliminary data in order to develop an individualized, patient-centred, therapy concept.

**Supplementary Information:**

The online version contains supplementary material available at 10.1186/s12885-022-10101-9.

## Background

MM is the third most common haematological malignancy worldwide with an age-dependent probability of occurrence [1]. As a consequence of a global aging population, the number of cases of MM is rising [1]. Symptoms of MM are multiform [2] and studies revealed that myeloma patients experience more symptoms and problems than patients with other haematological cancers [3]. Fortunately, survival rates are steadily increasing due to new diagnostic and therapeutic approaches [4–6]. The therapeutic goal is to achieve a deep remission while at the same time optimizing symptom control and maintaining the best possible quality of life (QoL). However, studies demonstrated that short and long-term impairments often interfere with regaining full functional capacity [7]. In summary, the HRQoL becomes more important in clinical decision making as currently there is not a satisfactory study situation in this respect [8].

Therefore, the aim of this study is to identify the most affected aspects of QoL before, during and after the different therapies of MM, and to detect influencing factors that can be taken into account when creating patient specific therapies, to enable the highest rate of QoL and to provide a basis for clinical decision making.

## Methods

### Study design, participants, procedures and questionnaires

For this prospective observational study, 70 patients with MM were recruited, from April 2013 until January 2015, prior to the start of treatment at the University Hospital Wuerzburg, regardless of the individual time point of primary diagnosis. The patients were either newly diagnosed or had previously received treatment. Inclusion criteria were age ≥ 18 years, histologically proven MM, and an informed written consent. Exclusion criteria was a prior diagnosis of a second malignancy, with the exception of basalioma. The study was approved by the Medical Faculty Local Ethics Committee of the University of Wuerzburg (vote-no 301/12).

During the inclusion interval, after detailed explanation of the interview procedure, consent form, and data protection, written informed consent was obtained from each patient with an indication of first-line or relapse treatment of MM. Consent could be withdrawn at any time during and after the survey. The patient population (***n = ***70) was divided into subgroups depending on first- or subsequent treatment and treatment concept (Table 1).

**Table 1:** Subgroups dependent on type of treatment

A First-line treatment	A1 Patients unsuitable for	
(*n* = 36, 51.4%)	SCT/high-dose chemotherapy (*n* = 6, 8.6%)	
	all obtained novel agent based therapy	
	A2 Patients qualifying for	A2a High-dose chemotherapy with
	transplant based concept (*n*=30, 42.8%)	autologous SCT (*n* = 9, 12.9%)
		A2b High-dose chemotherapy with
		tandem-SCT (*n* = 21, 30.0%)
		auto/auto (*n* = 18, 25.7%)
		auto/allo (*n* = 3, 4.3%)
		A2c High-dose chemotherapy with
		allogeneic SCT (*n* = 0)
B Higher treatment line	B1 Patients unsuitable for	
(*n* = 34, 48.6%)	SCT/high-dose chemotherapy (*n* = 18, 25.7%),	
	all obtained novel agent based therapy	
	B2 Patients qualifying for	B2a High-dose chemotherapy with
	transplant based concept (*n* = 16, 22.9%)	autologous SCT (*n *= 8, 11.4%)
		B2b High-dose chemotherapy with
		tandem-SCT (*n* = 4, 5.7%)
		auto/auto (*n* = 3, 4.3%)
		auto/allo (*n* = 1, 1.4%)
		B2c High-dose chemotherapy with
		allogeneic SCT (*n* = 4, 5.7%)

The first assessment was before the start of therapy. Patients without a transplant-based concept (SCT) were interviewed for the second time in a period of four weeks after the end of therapy. In the case of SCT, the second assessment was four weeks after the end of induction, but prior to the (first) SCT. For the entire study population, the last assessment was three months after the end of therapy. With regard to specific aspects of HRQoL in patients with MM, we used the “Quality of Life Questionnaire-Core 30” of the European Organization for Research and Treatment of Cancer (EORTC QLQ-C30) and the Multiple Myeloma Module (QLQ-MY20) [9].

In addition, the Ultra-Brief Screening Scale for Anxiety and Depression (4-Item Patient Health Questionnaire, PHQ-4) and a sociodemographic questionnaire were employed at the first assessment to gather information about suspected influencing factors of QoL. The PHQ-4 is a reliable and validated self-report questionnaire that consists of a 2-item depression scale (PHQ-2) [10, 11] and a 2-item anxiety scale (GAD-2) [12]. Each item is scored from 0 to 3 with a composite PHQ-4 total score ranging from 0 to 12 [13]. Scale scores ≥ 6 indicate moderate psychological distress and an increased risk for a depressive or an anxiety disorder, while scores > 9 indicate a severe psychological distress [13]. A score of 3-or-greater on both the depression and anxiety subscales has been shown to represent a reasonable cut-point for identifying at risk patients [10–12]. Therefore, we decided to focus specifically on those patients with a score of 6 or greater.

Sociodemographic data was assessed by a standardized questionnaire. It included information on nationality, age, gender, marital status, education level, occupation status, sick leave, occupational disability, and income. The questionnaires were paper-based, filled in either during a visit or at home.

Disease and treatment details as well as clinical information (e.g. lab values, performance status, comorbidities, last response to treatment) were extracted from medical records at defined time points. For better comparability with other studies, combination therapies were listed by Bortezomib-, Lenalidomide- and alkylating agent-based treatments. While participation in the clinical trial was documented, there was no pre-specified stratification of outcomes with respect to treatment within a trial.

### Statistical analysis

Sample size calculation was carried out by the G*Power Program. Data were analysed using SPSS Statistics software Version 25. First, for the descriptive analysis, quantitative variables (like haemoglobin level) were characterized by using means and standard deviations, medians, first and third quartiles, minimums and maximus. Qualitative variables (like sex, symptoms and academics) were analysed using a contingency table. For all variables, missing data was reported.

In the case of small size of subgroups, the procedure was descriptive. For all other subgroups, Chi-square test, t test, Mann–Whitney U test or Fisher’s-exact-test were used to check the influence of supposed risk factors for anxiety and depression and differences in patient characteristics in the different subgroups (patients with primary diagnosis or relapse etc.).

For the analysis of EORTC QLQ-C30 and MY20 normality was tested by q-q-plots and control variables were identified by the Mann–Whitney U test. Multilevel models (also known as linear mixed-effect models) were used for testing significance.

In the case of normal distribution, the t-test was used to investigate the influence of participating in clinical trials, of anxiety/depression and of gender on HRQoL. For other distributions, the Mann–Whitney U test was performed. In the case of normal distribution, mixed ANOVA analysis was performed to explore the change of HRQoL of this clinical-trial-group over the time. Without normal distribution, a group comparison was carried out by calculating differences between the last and the first assessment and checking those difference values by the Mann–Whitney U test. In the case of normal distribution, ANOVA analysis was used to investigate the influence of age (three groups ≤ 59, 60–69 and ≥ 70 years) on HRQoL, otherwise the Kruskal–Wallis test was perfomed.

Due to the small sample size, testing normality was abandoned and the Mann–Whitney U test was used directly for the analysis of the influence of anxiety and depression on HRQoL (for the whole study population and the subgroup with tandem-SCT over the assessment time).

The significance level for the whole statistical analysis was *p* = 0.05.

## Results

### Baseline patient and disease characteristics

The patient population (***N = ***70) was divided into subgroups depending on first- or higher-treatment-line and suitability for autologous/allogeneic/tandem-SCT (Table 1).

Twenty-four of the Sixty-five patients were participants of a clinical trial. 18 patients were excluded during the assessment. A detailed review of patient sociodemographic data, clinical data on comorbidities, symptoms, ECOG performance status and treatment history can be found in Supplemental material Table 1.

### MM treatment

Consistent with current therapy guidelines, most patients with newly diagnosed MM in this study were treated with a tandem-SCT (58,3% of first-line patients) and most patients with relapsed MM were treated with a novel agent-based therapy without SCT (52.9% of relapsed patients). Overall, bortezomib-containing treatment was the most common therapy (67.1% of all), followed by Lenalidomide-containing protocols (55.7% of all). Before start of therapy, 10% of all patients received radiotherapy with a decreasing number in the systemic treatment process. Treatment with bisphosphonates increased after the start of chemotherapy (from 24.3% to 40.6%).

Most patients with relapsed MM had previously received an intensive therapy. In average they had received 3.2 prior therapies (range 1–9, Supplementary Table 1).

### Anxiety and depression (PHQ-4)

The screening for anxiety and depression by using the PHQ-4 questionnaire as part of the first assessment, showed an average value of the 70 interviewed persons of 3.27 (minimum 0.00 and maximum 12.00), which corresponds to a low-grade risk increase compared to a reference population without MM [14].

There was no significant difference in anxiety and depression of patients with first-line or relapse treatment regarding severe (*p* = 0.358) or moderate psychological distress (*p* = 0.768). Moreover, if considering only those patients with a score ≥ 6, there was no significant difference in age, gender, marital status, intellect, stage at diagnosis (ISS), number of symptoms and clinical findings or time since diagnosis for all patient subgroups.

### HRQoL by treatment and over the course of time

All results regarding HRQoL are represented schematically in detail in Supplementary Table 2 and in summary in Supplementary Table 3. On average, first-line patients reported significantly lower pain level and better global health status, and even showed further improvement in these variables over time. Although there was no significant difference, most of the other parameters of HRQoL were perceived more favourably by patients with first-line treatment in comparison to patients with recurrent MM. In contrast, patients with relapsed MM presented with decreasing global health status and increasing pain, while they reported a significantly superior role functioning at baseline.

**Fig. 1:**
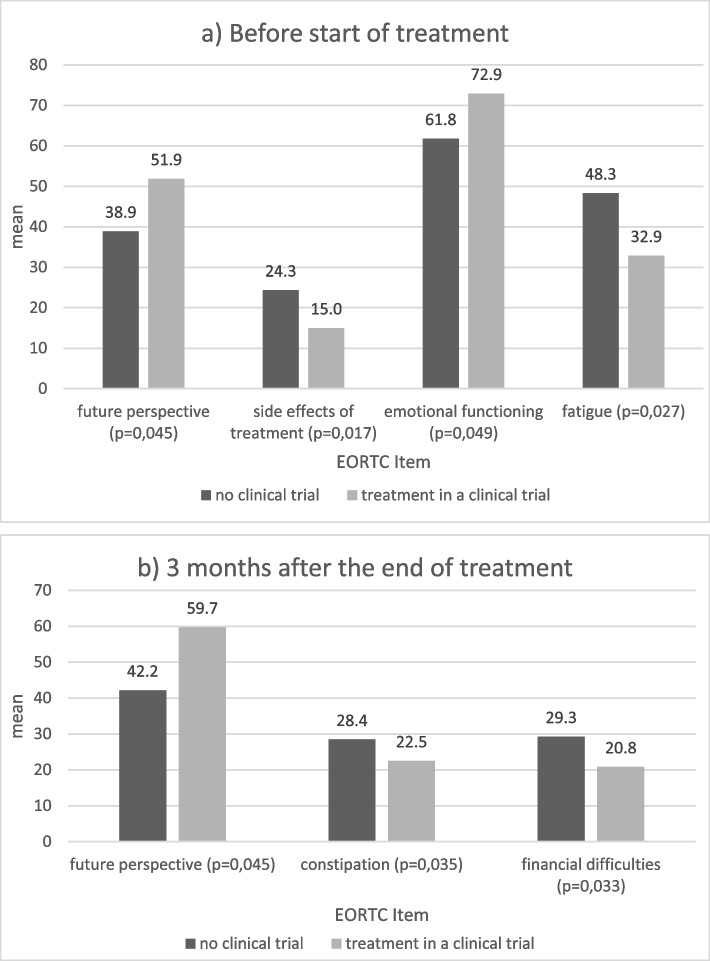
Significant influence of participation in clinical trials on HRQoL. **a** Before start of treatment. **b** 3 months after the end of treatment

In the parameters of body image, side effects of treatment, and cognitive functioning, the HRQoL of all patients decreased significantly after the start of chemotherapy. Patients with tandem transplantation did not perform significantly worse (Supplementary Table 2). Participation in clinical trials was correlated to a better HRQoL in numerous parameters at all time points compared to non-study patients, which was particularly evident in first-line patients (Fig. 1a and b).

### HRQoL by patient characteristics – gender and age

**Fig. 2:**
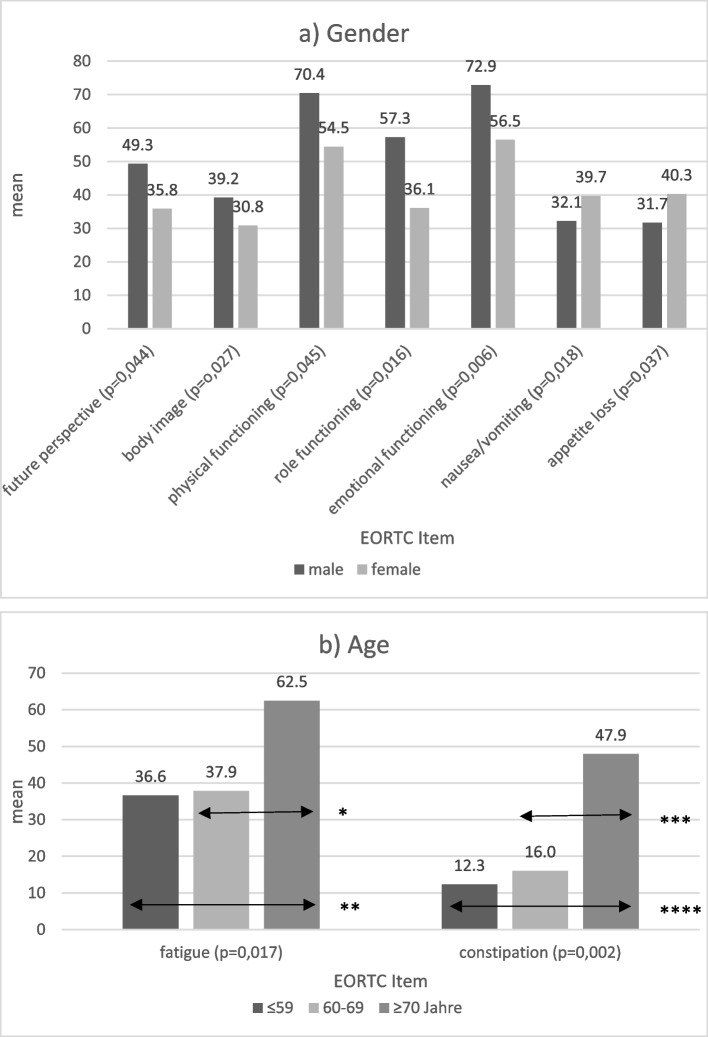
Significant influence of **a** gender and **b** age on HRQoL before start of treatment, *p = 0.034, **p = 0.024, *** *p* = 0.003, ****p = 0.001

Male patients indicated a better HRQoL in most parameters than women before start of treatment. As such, they reported a better future perspective, physical, role and emotional functioning, body image, less nausea/vomiting and loss of appetite (Fig. 2a).

Although better scores in younger patients (< 70 years) with MM suggest a better HRQoL before start of treatment, most parameters did not reach a significance level. None the less, these patients reported significantly less fatigue and constipation than elderly patients (Fig. 2b).

### HRQoL by anxiety and depression (PHQ-4)

**Fig. 3:**
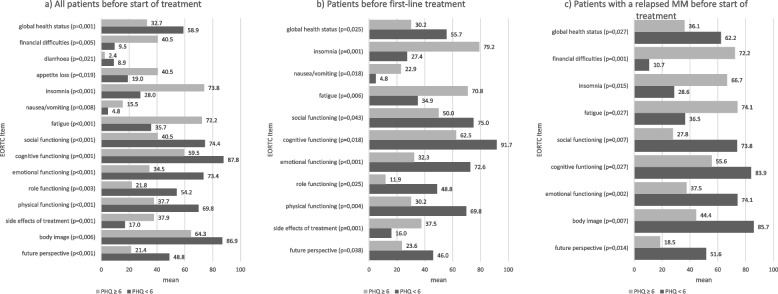
Significant influence of anxiety/depression on HRQoL before start of treatment. **a** All patients. **b** Patients with first-line treatment. **c** Patients with therapy of a relapsed MM

As Fig. 3 shows, anxiety and depression can have a significant impact on quality of life, with less anxious and depressed patients (especially PHQ < 6) scoring better on almost all parameters and at all measurement time points, regardless of the respective subgroups (Fig. 3a, b and c).

This was especially true for the first line patients (Fig. 3b), with only this group having significantly less side effects, a better role and physical functioning, for example. The effects of a PHQ < 6 over time are listed in Supplementary Table 2, where the group of first-line patients again reported less side effects and the least insomnia long term.

In contrast, only relapsed patients with a PHQ < 6 reported a better body image over the entire course of treatment and less financial difficulties up to 3 month after the end of treatment than anxious/depressive patients in this subgroup (Fig. 3c).

Considering the subgroup of the 46 patients who underwent transplant-based concepts, a PHQ < 6 was also associated with better HRQoL scores over time, with significantly less correlation in the tandem transplant group (Supplementary Table 2). In particular, the subgroup of patients with a single autologous SCT and PHQ < 6 in general suffered significantly less from disease symptoms (*p* = 0.005) and less from pain (0.012) as compared to patients with a PHQ ≥ 6 in this subgroup.

## Discussion

A central aspect of this study was to identify the most affected aspects of QoL during the course of treatment within defined patient subgroups, as well as to detect potential influencing factors of HRQoL in patients with MM. Under the presumption that anxiety and depression in patients with MM could be an important and common risk factor for reduced HRQoL in this situation [15–17], we decided to investigate the PHQ-4, which proved to be a suitable screening tool that was well accepted by patients. With an average value of 3.27, it showed an elevated risk for anxiety and depression in patients with MM, while data from a large sample of primary-care patients (without MM) showed a mean PHQ-4 score of 2.5 [11]. Patients with less anxiety and depression (especially PHQ < 6) indicated a better HRQoL in almost all parameters, at all assessment times, and regardless of patient subgroups. In particular, within the subgroup of first-line patients, fewer side effects were reported, including, for example, less insomnia up to 3 months after the end of treatment. Patients with relapsed myeloma and PHQ < 6 were significantly less concerned about financial aspects compared to the patients with elevated scores for anxiety and depression in this subgroup. Screening with a focus on anxiety and depression should therefore be performed early and repeatedly since an increased level of anxiety and depressiveness can have far-reaching effects on the further course of HRQoL. In particular, patients with PHQ ≥ 6 should be offered intensive psycho-oncological support at an early stage.

Matching the HRQoL data from the randomized phase III ALCYONE trial, showed an increasing global health status and decreasing pain under therapy in patients with newly diagnosed multiple myeloma ineligible for SCT [18]. This study was able to demonstrate the same effects for the subgroup of first-line patients. In contrast, we could detect opposing results in the relapse group. Most patients reported an impairment in the majority of the HRQoL parameters, especially more side effects over time with a slow recovery as the distance from therapy increased. This is consistent with the results that Dhir et al*.* presented at the last ASH Annual Meeting regarding SCT patients as well as the large population-based Canadian trial from Ebraheem et al. [19, 20]. However, in contrast to the Ebraheem study, which included only first-line patients eligible for SCT, we tried to also focus on the differences between patients in first-line therapy and relapsed MM patients. Although results did not reach a significance level, we determined a trend towards better performance in almost all aspects of HRQoL in patients under first-line treatment. This trend could be caused by the long time from diagnosis and associated multiple pre-therapies, which reference data largely supports [21, 22]. Even though we were able to include only 25 patients treated with tandem-SCT, these first results seem to be in line with the results of other research groups. Uyl‐de Groot et al*.* could demonstrate, that in a group of 51 patients, tandem-SCT was being subjectively well tolerated by the majority of patients with a relatively short duration of declined quality of live [23]. Therefore, especially for patients who worry about their QoL regarding to this intensive therapy, this could be an informative decision support for therapy modality.

In addition, this study identified age and gender as possible influence factors for multiple aspects of HRQoL in patients with MM. These results are largely consistent with the reference data [24–26]. Women seem to suffer more from physical restrictions, impaired emotional functioning, and reduced QoL before start of treatment. Elderly patients in our study reported significantly more fatigue and constipation. Mian et al*.* reported data from a cohort of 40 elderly patients (> 65 years), who underwent stem-cell transplantation and remained stable with regards to function and overall QoL whereas the mental health of the study participants had actually improved 6 months after the end of therapy [27]. If these observations were to be confirmed in larger studies, female and elderly patients might benefit from intensive and more specific supportive care in order to be able to better withstand intensive therapy concepts.

To the best of our knowledge, this is the first study, which post-hoc documented observed improvement in many HRQoL parameters in clinical trial participants compared to those treated outside a clinical trial. This could be due to good information given about the disease and treatment and possibly a closer connectivity to the therapeutic team. Future perspective, side effects of treatment, emotional functioning and fatigue were reported significantly better before the start of a treatment and persisted throughout the treatment timeframe. However, clinical trials have inclusion criteria, which could have led to differences in patient characteristics. Therefore, it is difficult to make a comparison of MM patients treated in clinical trials with patients treated within standard therapy concepts. Moreover, there was no explicit stratification of outcomes with respect to treatment within a trial. Nevertheless, these results should be taken into consideration in the therapy setting of patients with chronic diseases, who don’t meet inclusion criteria or don’t want to take part in clinical trials. In addition, these patients should also get intensive support from the help of artificial intelligence or special coaching programs, for example. There is starting to be documented evidence for the effectiveness of these forms of intensive support [28, 29].

As noted in the following section, this study has some limitations. The context of the investigation as a pilot study, at this stage, must be considered when interpreting the results. Due to the heterogeneous therapy groups, each cohort is very small and therefore the consequences are limited. Due to the small number of participants treated without novel agents (Supplementary Table 4 and 5), follow up studies are needed as a final position to HRQoL under the treatment by novel agents in comparison to conventional chemotherapy could not be taken. Additionally, we could not draw a comparison between autologous/autologous or autologous/allogenic tandem transplantation concepts. Furthermore, the median age of patients treated with autologous SCT was younger compared to other treatment groups. Therefore, any consequences taken from these results should be interpreted carefully. Finally, we only performed a single screening by PHQ-4 before the start of treatment. Instead of a single screening, patients with MM should be screened regularly for anxiety and depression to be able to provide early psycho-oncological support to improve physical and emotional functioning as well as general QoL.

## Conclusions

This study showed the importance of regular longitudinal assessments of PROs like anxiety and depression and QoL in routine clinical care of patients with MM before, during and after systemic therapy and also indicated that age and gender, as well as participation in a clinical trial may be important parameters to consider in clinical care treatments. Patients under treatment of relapsed myeloma were more often confronted with reduced QoL in different parameters, while first-line patients reported significant improvement in global health status and especially pain during therapy.

As the field moves towards an individualized, patient-centred therapy, concepts suggested for future studies include, for example, analysing artificial intelligence and coaching programs to accompany our patients.

## Supplementary Information

**Additional file 1: Supplementary Table 1.** Sociodemographic and clinical characteristics of the 70 patients with MM included in the study. **Supplementary Table 2.** *P*-values and mean-values. **Supplementary Table 3.** Summary of the most important results. **Supplementary Table 4.** Therapy of patients without stem cell transplantation. **Supplementary Table 5.** Therapy of patients with stem cell transplantation.

## Data Availability

The datasets generated, used and analysed during the current study are available from the corresponding author on reasonable request.

## Abbreviations

ANOVA: Analysis of variance
CRAB: Calcium level, renal dysfunction, anaemia and destructive bone lesions
DSMM: Deutsche Studiengruppe Multiples Myelom
ECOG: Eastern Cooperative Oncology Group
EORTC QLQ-C30: European Organization for Research and Treatment of Cancer (EORTC) quality of life questionnaire C30
EORTC QLQ-MY20: European Organization for Research and Treatment of Cancer (EORTC) myeloma-specific quality of life questionnaire MY20
GAD-2: Generalized Anxiety Disorder-2
HRQoL: Health-related quality of life
ISS: International Staging System
LDH: Lactate dehydrogenase
MM: Multiple Myeloma
PHQ-4: Patient Health Questionnaire-4
PRO: Patient reported outcome
QoL: Quality of life
SCT: Stem cell transplantation
