# Supplementary material for: The influence of baseline characteristics, treatment and depression on health-related quality of life in patients with multiple myeloma: a prospective observational study
Source: BMC Cancer. 2022 Oct 3;22:1032. doi: 10.1186/s12885-022-10101-9 (PMC9528097; doi:10.1186/s12885-022-10101-9)
Supplement: Supplementary file 1 — Additional file 1: Supplementary Table 1. Sociodemographic and clinical characteristics of the 70 patients with MM included in the study. Supplementary Table 2. P-values and mean-values. Supplementary Table 3. Summary of the most important results. Supplementary Table 4. Therapy of patients without stem cell transplantation. Supplementary Table 5. Therapy of patients with stem cell transplantation. [file 12885_2022_10101_MOESM1_ESM.docx]

# Supplemental material
